# Differential isoform expression of Allergin‐1 during acute and chronic inflammation

**DOI:** 10.1002/iid3.739

**Published:** 2022-11-25

**Authors:** Ruben J. Geerdink, Maria Inês Pascoal Ramos, Luuk L. van den Hoogen, Timothy R. D. J. Radstake, Shiro Shibayama, Akira Shibuya, Louis Bont, Linde Meyaard

**Affiliations:** ^1^ Center for Translational Immunology, University Medical Centre Utrecht Utrecht University Utrecht The Netherlands; ^2^ Department of Rheumatology and Clinical Immunology University Medical Centre Utrecht Utrecht The Netherlands; ^3^ Research Centre of Immunology, Tsukuba Institute ONO Pharmaceutical Co., Ltd. Tsukuba Ibaraki Japan; ^4^ Department of Immunology, Faculty of Medicine University of Tsukuba Tsukuba Ibaraki Japan; ^5^ Department of Paediatrics, Wilhelmina Children's Hospital University Medical Centre Utrecht Utrecht The Netherlands

**Keywords:** inhibitory receptor, neutrophils, nonclassical monocytes, respiratory syncytial virus, systemic lupus erythematosus

## Abstract

**Introduction:**

Neutrophils are crucial to antimicrobial defense, but excessive neutrophilic inflammation elicits immune pathology. Currently, no effective treatment exists to curb neutrophil activation. However, neutrophils express a variety of inhibitory receptors which may represent potential therapeutic targets to limit neutrophilic inflammation. Indeed, we previously showed that the inhibitory collagen receptor leukocyte‐associated immunoglobulin‐like receptor 1 (LAIR‐1) regulates neutrophilic airway inflammation and inhibits neutrophil extracellular trap formation. The inhibitory receptor Allergin‐1 is expressed by myeloid cells and B cells. Allergin‐1 suppresses mast cell and basophil activation, but a potential regulatory role on neutrophils remains unexplored. We aimed to demonstrate the regulation of neutrophils by Allergin‐1.

**Methods:**

We examine Allergin‐1 isoform expression on human neutrophils during homeostatic (healthy donors) and chronic inflammatory (systemic lupus erythematosus patients) conditions in comparison to other circulating leukocytes by flow cytometry. To reveal a potential role for Allergin‐1 in regulating neutrophilic inflammation, we experimentally infect wild‐type (WT) and Allergin‐1‐deficient mice with a respiratory syncytial virus (RSV) and monitor disease severity and examine cellular airway infiltrate. Flow cytometry was used to confirm Allergin‐1 expression by airway‐infiltrated neutrophils in RSV infection‐induced bronchiolitis patients.

**Results:**

Only the short 1 (S1) isoform, but not the long (L) or S2 isoform could be detected on blood leukocytes, with the exception of nonclassical monocytes, which exclusively express the S2 isoform. Allergin‐1 expression levels did not vary significantly between healthy individuals and patients with the systemic inflammatory disease on any interrogated cell type. Airway‐infiltrated neutrophils of pediatric RSV bronchiolitis patients were found to express Allergin‐1S1. However, Allergin‐1‐deficient mice experimentally infected with RSV did not show exacerbated disease or increased neutrophil airway infiltration compared to WT littermates.

**Conclusion:**

Allergin‐1 isoform expression is unaffected by chronic inflammatory conditions. In stark contrast to fellow inhibitory receptor LAIR‐1, Allergin‐1 does not regulate neutrophilic inflammation in a mouse model of RSV bronchiolitis.

## Graphical abstract

Allergin‐1 is a functional inhibitory receptor on mast cells and basophils, but its role on neutrophils has remained unexplored. Despite having the same downstream effectors (SHP‐1, SHP‐2) as functional inhibitory receptors on neutrophils (e.g., leukocyte‐associated immunoglobulin‐like receptor 1 and signal inhibitory receptor on leukocytes 1), Allergin‐1 does not appear to regulate neutrophilic inflammation in response to viral infection. These results highlight the functional diversity of inhibitory immune receptors.

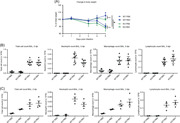

## 1 INTRODUCTION

Neutrophilic granulocytes are vital to antimicrobial defense and other immune functions. However, neutrophil effector mechanisms, such as reactive oxygen species production, the release of proteolytic enzymes, and neutrophil extracellular trap formation, damage host tissues as well as pathogens.^1^, ^2^ Excessive neutrophilic inflammation thereby induces immune pathology in conditions as diverse as a respiratory syncytial virus (RSV) infection‐induced bronchiolitis and ischemia‐reperfusion injury.^3^ By suppressing neutrophil activation, immune pathology may be prevented in a myriad of conditions. However, no effective treatment is currently available to limit neutrophilic inflammation.

Neutrophils express a multitude of inhibitory receptors that negatively regulate their activation status, by providing an activation threshold and/or counteracting activating signals.^4^, ^5^ The efficacy and potency of targeting inhibitory receptors for therapy have been demonstrated by checkpoint blockade immunotherapy in cancer.^6^ Possibly, activating inhibitory receptors to suppress excessive inflammation, rather than blocking inhibitory receptors to strengthen the antitumour response, may provide new avenues of treatment for neutrophil‐driven immune pathology.

Allergy inhibitory receptor 1 (Allergin‐1) is an immunoreceptor tyrosine‐based inhibitory motif (ITIM)‐bearing immunoglobulin (Ig)‐like receptor that is primarily expressed on B cells (in humans) and myeloid cells (in humans and mice), including all types of granulocytes.^7^ The inhibitory function of Allergin‐1 on mast cells and basophilic granulocytes and its regulatory role in allergic diseases has been demonstrated.^8^, ^9^, ^10^, ^11^, ^12^ A potential regulatory function of Allergin‐1 on neutrophilic granulocytes and monocytes has received less scrutiny, however. Since neutrophils highly express Allergin‐1 and are regulated by other ITIM‐bearing inhibitory receptors, such as leukocyte‐associated immunoglobulin‐like receptor 1 (LAIR‐1) and signal inhibitory receptor on leukocytes 1 (SIRL‐1), an inhibitory function of Allergin‐1 on neutrophils seems plausible.^13^, ^14^ Additionally, Allergin‐1 promotes the clearance of apoptotic debris by enhancing phagocytosis by macrophages, which suppresses autoantibody production.^15^

Human Allergin‐1 has three isoforms, namely Allergin‐1S1 (short form), Allergin‐1S2, and Allergin‐1L (long form), defined by the inclusion or exclusion of its two extracellular Ig domains. The S1 and S2 isoforms each contain one of the two Ig domains, whereas the L isoform contains both. Most attention has gone to the Allergin‐1S1 and/or Allergin‐1L isoforms, the former of which is homologous to the mouse Allergin‐1.^7^

In the current study, we investigate possible changes in Allergin‐1 isoform expression due to chronic systemic inflammation as well as regulation by Allergin‐1 of acute neutrophilic inflammation. In particular, we examine neutrophilic airway inflammation, since Allergin‐1 on mast cells has been reported to suppress allergic airway inflammation. We compare the isoform expression of Allergin‐1 on various leukocyte subsets in blood during homeostatic conditions and in chronic systemic inflammation, i.e., in healthy donors and systemic lupus erythematosus (SLE) patients. To examine a potential regulatory role for Allergin‐1 in acute neutrophilic airway inflammation, we employ an experimental mouse model of RSV‐induced bronchiolitis.

## 2 MATERIALS AND METHODS

### 2.1 Flow cytometry

The red blood cells in heparinized venous blood samples obtained from healthy donors and SLE patients were lysed with an ammonium chloride buffer (155 mM NH_4_Cl, 10 mM KHCO_3_, and 0.1 mM ethylenediaminetetraacetic acid). Erythrocytes in blood samples of pediatric RSV patients were lysed by treatment with hypotonic distilled water. Aspirated sputum samples of intubated pediatric RSV patients were resuspended in phosphate‐buffered saline (PBS) solution and passed through 100‐µm‐pore filters twice to obtain a single‐cell suspension. Erythrocyte‐depleted single‐cell suspensions were incubated with viability dye followed by fluorochrome‐conjugated antibodies and analyzed on an LSR Fortessa (BD Biosciences). Acquisitions were analyzed using FlowJo software (version 10.0.7, Treestar). See Supporting Information: Figure 1 for the employed gating strategy and Supporting Information: Table 1 for an overview of the antibodies used in this study. For an overview of the origin of samples, in which experiments they were used, and in which Figures their data is presented, see Supporting Information: Figure 2.

### 2.2 Animals

*Mirl1* ^−/−^ mice were generated on the BALB/c background by Hitomi et al.^7^ *Mirl1* ^−/−^ mice and their wild‐type (WT) littermates were bred under specific pathogen‐free conditions at the animal facility of Utrecht University. All animal studies were approved by the Institutional Animal Care and Use Committee and carried out in accordance with national and institutional guidelines.

### 2.3 Mouse RSV infection

An 8–12‐week‐old female BALB/c *Mirl1* ^−/−^ mice or their WT littermates were intranasally inoculated with 1 × 10^7^ PFU of RSV‐A2 in 50 μl of PBS. RSV‐A2 preparation, quantitative assay for RSV‐A2 titration, and RSV‐A2 infection of mice, including, intranasal inoculation, termination, and sample collection, were performed as described previously. Mice were killed on Day 2 or 5 postinfection.^14^ For the quantification and differentiation of airway‐infiltrated cells whole‐lung bronchoalveolar lavage (BAL) was performed using 1.0 ml of PBS. Cells obtained by BAL were quantified using a hemocytometer and differentiated based on morphology following Giemsa staining.

### 2.4 Healthy donors and patients

Peripheral venous blood was obtained from healthy adult volunteers (*n* = 8), SLE patients (*n* = 13), and pediatric RSV bronchiolitis patients (*n* = 3). Aspirated sputum from intubated, mechanically ventilated RSV bronchiolitis patients was obtained by instillation and reaspiration of physiological saline in the endotracheal tube. The infants were aged less than 1 year and had polymerase chain reaction‐proven RSV bronchiolitis, but were otherwise healthy with no detectable coinfections. All subjects or their caretakers gave written informed consent and protocols were approved by the institutional review board.^13^

### 2.5 Statistical analyses

To determine the statistical significance of differences in Allergin‐1 isoform expression between healthy donors and SLE patients, the unpaired Mann–Whitney *U* test was used. The Kruskal–Wallis test was employed to determine whether there were statistically significant differences between the four groups of mice (WT and *Mirl1* ^−/−^ with PBS or RSV) in the RSV infection model.

## 3 RESULTS AND DISCUSSION

### 3.1 Differential expression of Allergin‐1S1 and Allergin‐1S2 isoforms on healthy blood leukocytes

**Figure 1:**
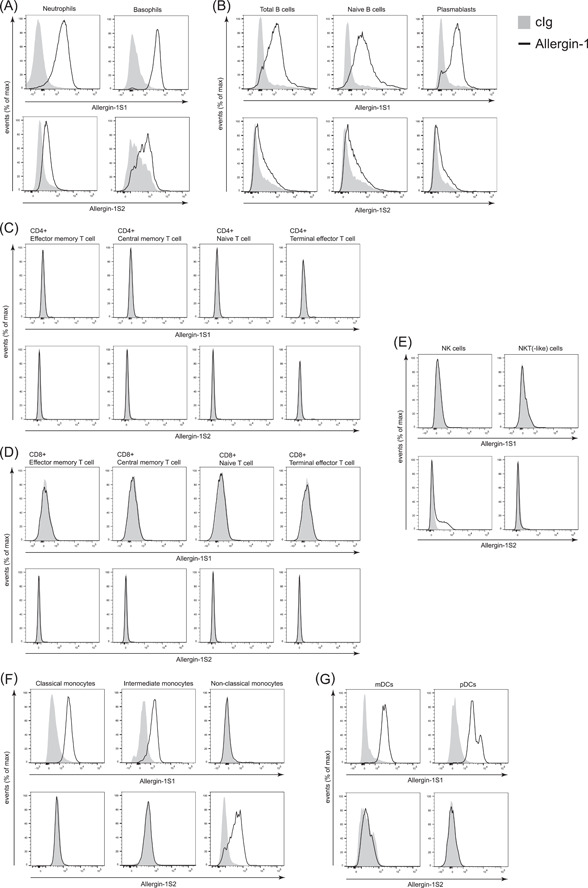
Differential expression of Allergin‐1 isoforms on blood leukocytes of healthy donors. Blood leukocytes obtained from healthy donors (*n* = 8) were stained for cell lineage markers and Allergin‐1S1 and Allergin‐1S2 isoforms. All leukocyte populations were gated based on forward‐ and side‐light scatter. Doublets and nonvital cells were excluded from the analysis. Black lines indicate Allergin‐1 expression and gray plots represent isotype‐matched control immunoglobulin g (cIg); representative plots are shown. Leukocyte subsets were identified as follows: (A) neutrophils, CD14− CD16+, basophils CD14− CD16− CD117− FcεRI+; (B) total B cells, CD19+, naïve B cells, CD19+ CD27− IgD+, plasmablasts, CD19+ CD27+ IgD−; (C) CD4+ effector memory T cells, CD3+ CD4+ CD27− CD45R0+, CD4+ central memory T cells, CD3+ CD4+ CD27+ CD45R0+, CD4+ naïve T cells, CD3+ CD4+ CD27+ CD45R0−, CD4+ terminal effector T cells, CD3+ CD4+ CD27− CD45R0−; (D) CD8+ effector memory T cells, CD3+ CD8+ CD27− CD45R0+, CD8+ central memory T cells, CD3+ CD8+ CD27+ CD45R0+, CD8+ naïve T cells, CD3+ CD8+ CD27+ CD45R0−, CD8+ terminal effector T cells, CD3+ CD8+ CD27− CD45R0‐; (E) natural killer (NK) cells, CD3− CD56+, NKT‐like cells, CD3+ CD56+; (F) classical monocytes, CD3− CD19− CD56− HLA‐DR+ CD14++ CD16−, intermediate monocytes, CD3− CD19− CD56− HLA‐DR+ CD14+ CD16+, nonclassical monocytes, CD3− CD19− CD56− HLA‐DR+ CD14− CD16++; and (G) myeloid dendritic cells (mDCs), CD3− CD19− CD56‐ HLA‐DR+ CD11c+ BDCA1+, plasmacytoid dendritic cells (pDCs), CD3− CD19− CD56− HLA‐DR+ CD11c− BDCA2+. See Supporting Information: Figure 1 for the employed gating strategy. IgD, immunoglobulin D; NKT, natural killer T.

We determined the expression of the S1 and S2 isoforms of Allergin‐1 on the blood leukocytes of healthy volunteers by flow cytometry. Numerous leukocyte subsets were interrogated, namely: neutrophils and basophils (Figure 1A); naïve B cells and plasmablasts (Figure 1B); both CD4 + and CD8 + naïve, central memory, effector memory, and terminal effector T cells, natural killer (NK) cells, and natural killer T (NKT) cells (Figure 1C–E); and classical, intermediate, and nonclassical monocytes, myeloid dendritic cells (mDCs), and plasmacytoid dendritic cells (pDCs) (Figure 1F,G). For gating strategies see Supporting Information: Figure 1.

Allergin‐1S1 was highly expressed on blood neutrophils and basophils, whereas Allergin‐1S2 was expressed at low or undetectable levels (Figure 1A). Similar results were obtained for B cells, with notable Allergin‐1S1 expression on naïve B cells and plasmablasts, but minor or nonexistent Allergin‐1S2 expression. Differentiation of naïve B cells into plasmablasts did not appear to affect the expression of Allergin‐1 isoforms (Figure 1B). None of the assayed CD4+ and CD8+ T lymphocyte subpopulations expressed either Allergin‐1S1 or Allergin‐1S2 (Figure 1C,D). Among innate lymphocytes, however, there appeared to be a subpopulation of NK cells that expressed Allergin‐1S2 (Figure 1E), this population was not defined by high CD56 expression (data not shown). NKT(‐like) cells did not express Allergin‐1 (Figure 1E). The expression of Allergin‐1 on both pDCs and mDCs was restricted to the S1 isoform (Figure 1G).

We detected expression of Allergin‐1S1, but not Allergin‐1S2, on classical and intermediate monocytes (Figure 1F). In contrast, Allergin‐1S2, but not Allergin‐1S1, was detected on nonclassical monocytes. Differentiation of classical monocytes into nonclassical monocytes coincided with the loss of Allergin‐1S1 and the upregulation of Allergin‐1S2. On the whole, the expression of the S1 and S2 isoforms appeared to be largely mutually exclusive on all cell types. In fact, Allergin‐1S2 could only be conclusively detected on nonclassical monocytes. Since surface expression of the L isoform would result in simultaneous detection of both the S1 and S2 Ig domains, this rules out the in vivo expression of the L isoform on blood leukocytes during homeostasis. In contrast, the initial identification of Allergin‐1 was made by the detection of the complementary DNA (cDNA) for Allergin‐1L in a cDNA library derived from the human bone marrow stromal cell line HAS303.^7^ Possibly, Allergin‐1L is not expressed by blood leukocytes, but is expressed by cells in the bone marrow or other tissues.

Interestingly, classical monocytes expressed Allergin‐1S1 but lost this expression when they differentiated into nonclassical monocytes, which, in turn, started to express Allergin‐1S2. While the ligand for Allergin‐1 is currently not known, an isoform shift may also impact ligand interaction or specificity. The biological relevance of this change in Allergin‐1 isoform expression would be an interesting subject for further inquiry. Moreover, while neutrophils represent a homogeneous population where Allerin‐1 expression is concerned (Figure 1A), other cell types, including plasmablasts, pDCs, basophils, and NK cells (Figure 1B,E,G), comprise more heterogeneous populations. Possibly, this represents subpopulations within these cell types or differences in activation state that our current flow cytometry panel was unable to distinguish.

### 3.2 Expression of Allergin‐1 on blood leukocytes of SLE patients

Next, we assessed whether an in vivo inflammatory milieu would affect Allergin‐1 isoform expression. To this end, we investigated blood samples of patients with a systemic inflammatory disease, namely SLE. Neutrophils and B cells expressed Allergin‐1S1 (Figure 1A,B), and both are suggested to be intimately involved in the pathogenesis of SLE.^16^, ^17^ Moreover, by promoting the phagocytosis of apoptotic debris by macrophages, Allergin‐1 suppresses the production of autoantibodies, for example, against double‐stranded DNA, which is characteristic of SLE.^15^

**Figure 2:**
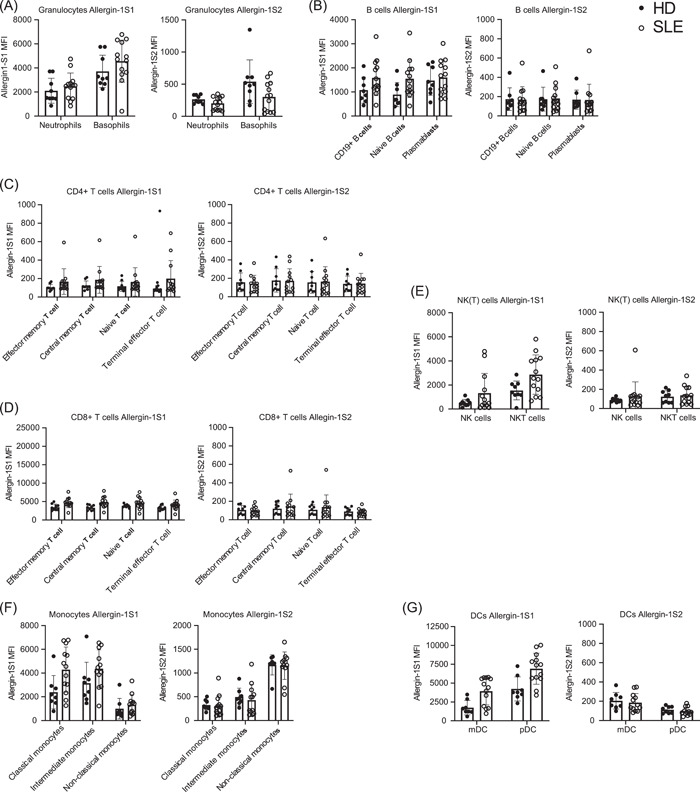
Allergin‐1 isoform expression on blood leukocytes of healthy donors and SLE patients. Blood leukocytes obtained from healthy donors (*n* = 8) and SLE patients (*n* = 13) were stained for cell lineage markers and Allergin‐1S1 and Allergin‐1S2 isoforms. All leukocyte populations were gated based on forward‐ and side‐light scatter. Doublets and nonvital cells were excluded from the analysis. Cell populations were identified as previously described; see also Supporting Information: Figure 1. Median fluorescence intensity (MFI) of the Allergin‐1S1 and ‐S2 isoforms on the following cell populations are shown: (A) neutrophils and basophils; (B) total B cells, naïve B cells, and plasmablasts; (C) CD4+ effector memory T cells, central memory T cells, naïve T cells, and terminal effector T cells; (D) CD8+ effector memory T cells, central memory T cells, naïve T cells, and terminal effector T cells; (E) NK cells and NKT(‐like) cells; (F) classical monocytes, intermediate monocytes, and nonclassical monocytes; and (G) myeloid dendritic cells (mDCs) and plasmacytoid dendritic cells (pDCs). There were no statistically significant differences (*p* < .05) as determined by the Mann–Whitney *U* test. DC, dendritic cell; NK, natural killer; NKT, natural killer T; SLE, systemic lupus erythematosus

Although there was a tendency for higher Allergin‐1S1 expression on, in particular, monocytes, mDCs, and pDCs from SLE patients, no statistically significant differences in Allergin‐1 isoform expression were observed between healthy donors and SLE patients (Figure 2). However, the heterogeneity of the SLE patient population may have obscured the fact that some individual patients expressed notably higher levels of Allergin‐1S1 (Figure 2A,G). Expression levels of Allergin‐1S2 were wholly comparable between the two populations (Figure 2). Additionally, while the absolute cell counts of SLE patients’ blood leukocytes and their relative cell type percentages may differ from healthy controls, for example, due to neutropenia, the percentage of Allergin‐1 isoform‐expressing cells among these cell types remained unchanged.

Similar to healthy donors, classical and intermediate monocytes of SLE patients expressed Allergin‐1S1 but did not express Allergin‐1S2, whereas nonclassical monocytes only expressed the Allergin‐1S2 isoform. Therefore, the L isoform was also not detected under systemic inflammatory conditions. Whether the Allergin‐1 expression in tissues is impacted remains to be determined.

### 3.3 Allergin‐1 does not limit RSV disease severity in mice

**Figure 3:**
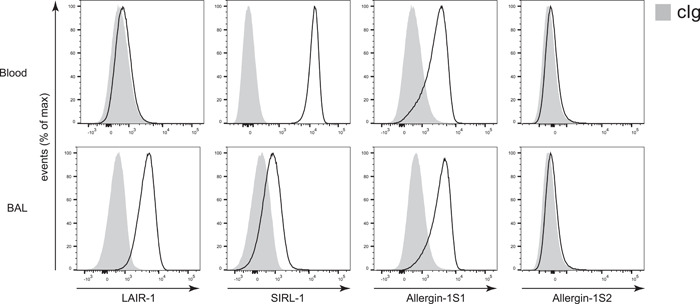
Allergin‐1S1 is expressed on both blood and airway‐infiltrated neutrophils of RSV bronchiolitis patients. Blood and aspirated airway‐infiltrated leukocytes obtained from intubated pediatric RSV bronchiolitis patients (*n* = 3) were stained for cell lineage markers, LAIR‐1, SIRL‐1, and the Allergin‐1S1 and Allergin‐1S2 isoforms. Gray plots represent isotype‐matched control immunoglobulin g (cIg); representative graphs are shown. BAL, bronchoalveolar lavage;LAIR‐1, leukocyte‐associated immunoglobulin‐like receptor 1; RSV, respiratory syncytial virus; SIRL‐1, signal inhibitory receptor on leukocytes 1.

An inhibitory function of Allergin‐1 on two types of granulocytes, namely mast cells and basophils, has previously been reported for allergic conditions, including anaphylaxis and allergic airway inflammation.^10^, ^11^, ^12^ To investigate a possible regulatory function of Allergin‐1 on neutrophilic granulocytes, we examined RSV infection, an infectious disease with prominent neutrophilic airway inflammation. We previously reported that inhibitory receptor expression patterns differ between blood and airway‐infiltrated neutrophils.^13^, ^14^ Therefore, we initially determined whether Allergin‐1 is expressed on the blood and airway‐infiltrated neutrophils of intubated pediatric RSV bronchiolitis patients by flow cytometry. Allergin‐1S1, but not Allergin‐1S2, was expressed by both blood and airway‐infiltrated neutrophils (Figure 3). Of note, we only enlisted three patients at a single time point for this part of the study (see Supporting Information: Figure 3 for the additional patients). We can, therefore, not exclude that Allergin‐1 expression may vary with disease severity throughout the course of the disease.

**Figure 4:**
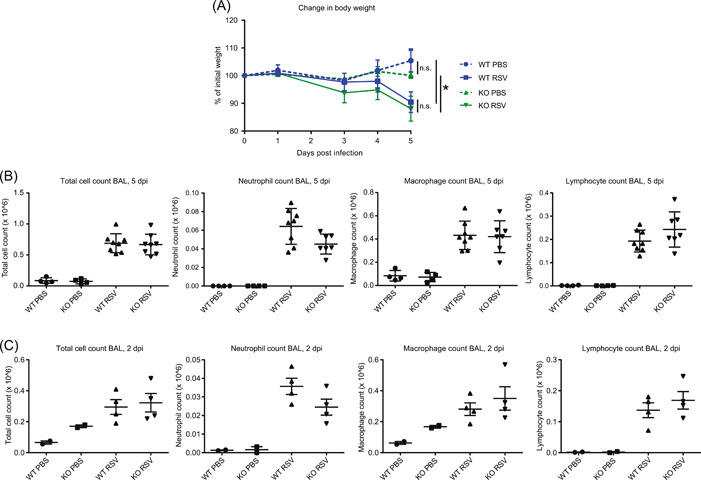
Allergin‐1 deficiency does not affect experimental RSV infection in mice. (A) BALB/c *Mirl1* ^−/−^ mice (KO) and wild‐type (WT) littermates inoculated with RSV (*n* = 8) or PBS (*n* = 4) were weighed daily; data are represented as weight change in percentage relative to Day 0. Terminal bronchioalveolar lavage (BAL) was performed on mice on Day 5 (B) and Day 2 (C) postinfection (dpi). Total cell counts and differential (neutrophil, macrophage, and lymphocyte) cell counts, based on cellular morphology, are shown. Data at 5 dpi are representative of two independent experiments, each with comparable group sizes (*n* = 8 for WT/KO RSV, *n* = 4 for WT/KO PBS); data at 2 dpi represent one experiment (*n* = 4 for WT/KO RSV, *n* = 2 WT/KO for PBS). Statistical significance was determined by the Kruskal–Wallis test. KO, knockout; ns, not significant; PBS, phosphate‐buffered saline; RSV, respiratory syncytial virus. *p < .05.

To determine a possible limiting role of Allergin‐1 in neutrophilic airway infiltration, we inoculated WT and Allergin‐1‐deficient (*Milr1* ^−/−^, knockout) BALB/c mice with RSV, or PBS as control, and followed weight loss as a marker of disease severity over time. Additionally, we assessed leukocyte airway infiltration by terminal BAL at 2 and 5 days postinfection (Figure 3B–D). RSV‐infected mice lost weight compared to control PBS‐inoculated mice, but no differences were observed between WT and *Milr1* ^−/−^ mice (Figure 4A). Experimental RSV infection resulted in leukocyte airway infiltration on both Day 2 and Day 5 postinfection. However, total cell counts, neutrophil counts, macrophage counts, and lymphocyte counts did not differ between WT and *Milr1* ^−/−^ mice (Figure 4B,C). Thus, Allergin‐1 did not limit disease severity or leukocyte airway infiltration in this experimental mouse model of RSV infection. However, we did not assess the role of Allergin‐1 in limiting viral replication by measuring viral loads in the lungs of the mice; this represents a limitation of our study. Unfortunately, a potential role for the Allergin‐1 isotype switch from S1 to S2 observed in human classical versus nonclassical monocytes could not be examined in our mouse model, since mice only express the S1 isoform.

By using LAIR‐1‐deficient mice in the same model of RSV disease, we previously demonstrated that the inhibitory collagen receptor LAIR‐1 plays a critical role in regulating neutrophil airway influx and limiting disease severity.^14^ Both LAIR‐1 and Allergin‐1 are ITIM‐bearing inhibitory receptors that recruit the downstream phosphatases SHP‐1 and SHP‐2 upon ligation. Nevertheless, their role in the regulation of neutrophil migration and/or activity appears to differ greatly. Possibly, the availability of the respective ligands of LAIR‐1 and Allergin‐1 in the lungs is responsible. While collagen(‐like) molecules are known ligands of LAIR‐1, the ligand of Allergin‐1 is unknown. However, airway hyperresponsiveness and inflammation in response to house dust mites are suppressed by Allergin‐1 on mast cells and lung‐resident CD11b+ dendritic cells, respectively.^10^, ^12^ This suggests that ligands for Allergin‐1 are present in lung tissue. Whether or not neutrophils can interact with these ligands in a manner that results in Allergin‐1‐mediated inhibition, remains to be determined. The development of agonists, for example, agonistic antibodies, or the identification of the ligand(s) of Allergin‐1 would facilitate further investigation into the function of Allergin‐1 on neutrophils.

In conclusion, we compared the expression of Allergin‐1 isoforms on human blood leukocytes in homeostatic and systemic inflammatory conditions and found no significant differences. A potential caveat, however, is that the SLE patient population is rather heterogeneous and we enlisted a limited number (*n* = 13) of patients. This may obscure individual differences or fail to reveal the existence of possible subpopulations with altered expressions. The Allergin‐1S1 isoform was expressed by every interrogated myeloid cell, as well as B lymphocytes. In contrast, the Allergin‐1L isoform could not be detected on any subset of blood leukocytes, and the S2 isoform was only detected on nonclassical monocytes after their transition from classical monocytes, which solely express the S1 isoform. While airway‐infiltrated neutrophils expressed Allergin‐1, genetic ablation of Allergin‐1 did not affect disease severity or leukocyte infiltration in a mouse model of RSV disease. The biological relevance of the Allergin‐1S1 to Allergin‐1S2 isoform switch on classical and nonclassical monocytes, respectively, as well as the inhibitory potential of Allergin‐1S1 on neutrophils, are prime targets for further investigation.

## AUTHOR CONTRIBUTIONS

Ruben J. Geerdink and Maria Inês Pascoal Ramos performed the research. Ruben J. Geerdink, M. Inês Pascoal Ramos, Louis Bont, and Linde Meyaard designed the research study. Luuk L. van den Hoogen, Timothy R. D. J. Radstake, Shiro Shibayama, and Akira Shibuya contributed essential reagents, patient material, and/or tools. Ruben J. Geerdink analyzed the data and wrote the paper. Louis Bont and Linde Meyaard contributed significantly to the writing of the paper. All authors read and approved the final manuscript.

## Supporting information


**Supplementary Figure 1. Gating strategy for immunophenotyping**. The gating strategy that was used to identify leukocyte subsets in the flow cytometry data is shown for a representative healthy donor. Leukocyte subsets were identified as follows: (A) neutrophils, CD14− CD16+, basophils CD14− CD16− CD117− FcεRI+; (B) total B cells, CD19+, naïve B cells, CD19+ CD27− IgD+, plasmablasts, CD19+ CD27+ IgD−; (C) CD4+ effector memory T cells, CD3+ CD4+ CD27− CD45R0+, CD4+ central memory T cells, CD3+ CD4+ CD27+ CD45R0+, CD4+ naïve T cells, CD3+ CD4+ CD27+ CD45R0−, CD4+ terminal effector T cells, CD3+ CD4+ CD27− CD45R0‐; (D) CD8+ effector memory T cells, CD3+ CD8+ CD27− CD45R0+, CD8+ central memory T cells, CD3+ CD8+ CD27+ CD45R0+, CD8+ naïve T cells, CD3+ CD8+ CD27+ CD45R0−, CD8+ terminal effector T cells, CD3+ CD8+ CD27− CD45R0−; (E) natural killer (NK) cells, CD3− CD56+, NKT‐like cells, CD3+ CD56+; (F) classical monocytes, CD3− CD19− CD56− HLA‐DR+ CD14++ CD16−, intermediate monocytes, CD3− CD19‐ CD56‐ HLA‐DR+ CD14+ CD16+, non‐classical monocytes, CD3− CD19− CD56‐ HLA‐DR+ CD14− CD16++; (G) myeloid DCs (mDCs), CD3− CD19− CD56− HLA‐DR+ CD11c+ BDCA1+, plasmacytoid DCs (pDCs), CD3− CD19− CD56− HLA‐DR+ CD11c− BDCA2+.

**Supplementary Figure 2. Overview of samples used in this study**. This figure illustrates the origin of the samples and the data generated with them as used in the current study. Also listed are the Figures in which the data is presented. Created with BioRender.

**Supplementary Figure 3. Allergin‐1 isotype expression on blood and airway neutrophil of two additional RSV bronchiolitis patients**. Blood and aspirated airway‐infiltrated leukocytes obtained from two intubated pediatric RSV bronchiolitis patients (in addition to the patient presented in Figure 3) were stained for cell lineage markers, LAIR‐1, SIRL‐1, and the Allergin‐1S1 and Allergin‐1S2 isoforms. Grey plots represent isotype‐matched control Ig (cIg).

Supporting information.

Supporting information.

Supporting information.

## Data Availability

The data that support the findings of this study are available from the corresponding author upon reasonable request.
