# Supplementary material for: Differential isoform expression of Allergin‐1 during acute and chronic inflammation
Source: Immun Inflamm Dis. 2022 Nov 25;10(12):e739. doi: 10.1002/iid3.739 (PMC9695092; doi:10.1002/iid3.739)
Supplement: Supplementary file 2 — Supplementary Figure 2. Overview of samples used in this study. This figure illustrates the origin of the samples and the data generated with them as used in the current study. Also listed are the Figures in which the data is presented. Created with BioRender. [file IID3-10-e739-s004.pdf]

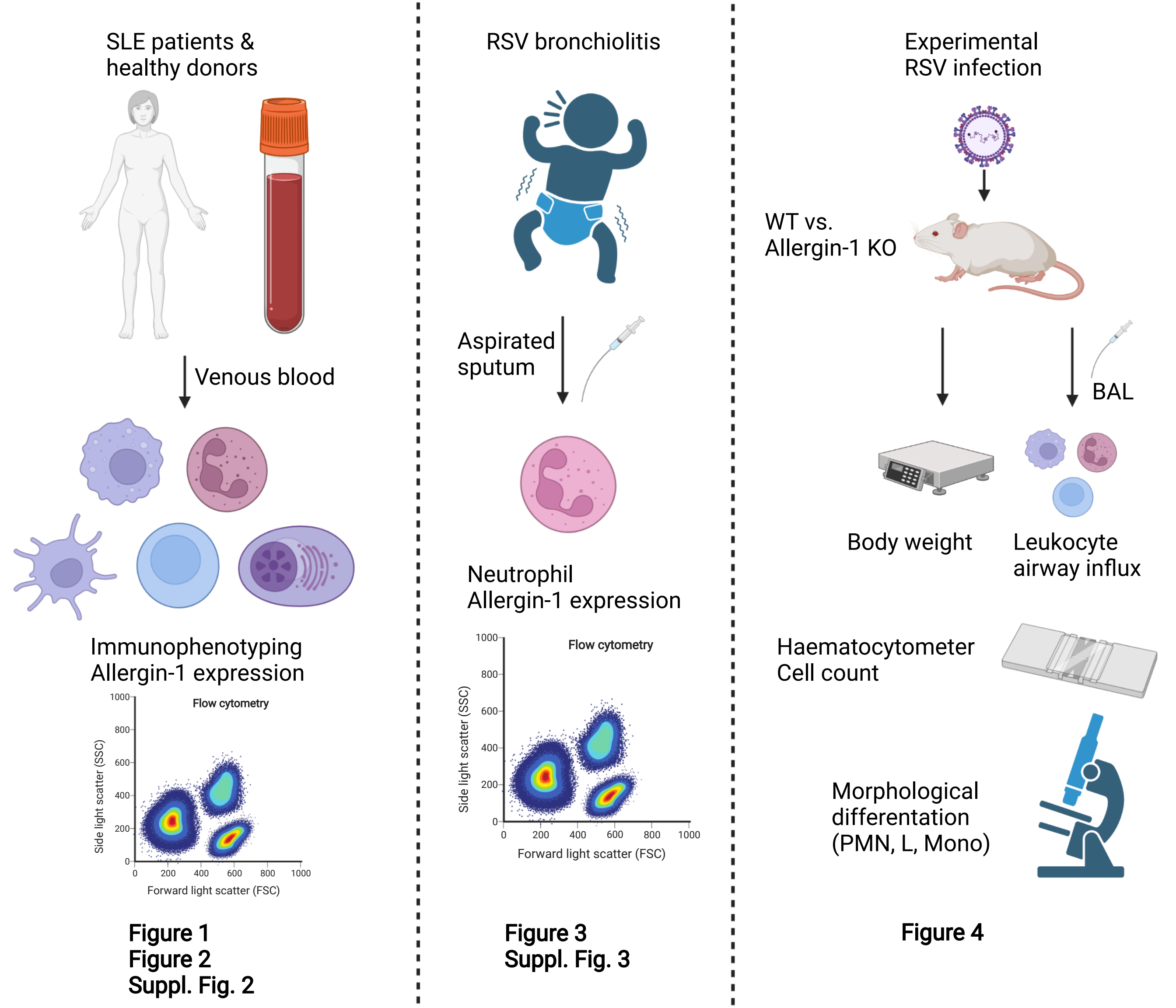

SLE patients & healthy donors
Venous blood
Immunophenotyping Allergin-1 expression
Flow cytometry
Side light scatter (SSC)
Forward light scatter (FSC)
Figure 1
Figure 2
Suppl. Fig. 2
RSV bronchiolitis
Aspirated sputum
Neutrophil Allergin-1 expression
Flow cytometry
Side light scatter (SSC)
Forward light scatter (FSC)
Figure 3
Suppl. Fig. 3
Experimental RSV infection
WT vs. Allergin-1 KO
BAL
Body weight
Leukocyte airway influx
Haematocytometer Cell count
Morphological differentation (PMN, L, Mono)
Figure 4
