# Supplementary material for: Differential isoform expression of Allergin‐1 during acute and chronic inflammation
Source: Immun Inflamm Dis. 2022 Nov 25;10(12):e739. doi: 10.1002/iid3.739 (PMC9695092; doi:10.1002/iid3.739)
Supplement: Supplementary file 4 — Supporting information. [file IID3-10-e739-s002.docx]

**Supplementary Table 1. Antibodies used in the study.**

This table compiles the antibodies used in the current study. Listed are the target of the antibody, its clone, the conjugated fluorochrome, and wherefrom it was purchased or by whom it was produced.

|  | **Target** | **Clone** | **Fluorochrome** | **Origin** |
| --- | --- | --- | --- | --- |
| **Primary A** | Control Ig rIgG2a | R35-95 (rIgG2a) | purified | BD Biosciences 553927 |
|  | Control Ig mIgG2a | G155-178 (mIgG2a) | purified | BD Biosciences 553454 |
|  |  |  |  |  |
| **Primary B** | Allergin-1S1 | EX32 (rIgG2a) | purified | ONO Pharmaceutical |
|  | Allergin-1S2 | EX29 (mIgG2a) | purified | ONO Pharmaceutical |
|  |  |  |  |  |
| **Secondary** | rat IgG | donkey polyclonal | AF647 | Jackson ImmunoResearch 712-605-153 |
|  | mouse IgG | donkey polyclonal | AF488 | Jackson ImmunoResearch 715-545-151 |
|  |  |  |  |  |
| **Panel 1** | CD19 | HIB19 | eF450 | eBioscience 48-0199-42 |
|  | CD27 | L128 | BV510 | BD Biosciences 563092 |
|  | IgD | 3G8 | AF700 | BD Biosciences 558122 |
|  | CD24 | ML5 | PerCP-Cy5.5 | BD Biosciences 561647 |
|  | CD38 | HIT2 | PE-Cy7 | eBioscience 25-0389-42 |
|  | CXCR3 | G025H7 | BV605 | BioLegend 353728 |
|  |  |  |  |  |
| **Panel 2** | CD3 | OKT3 | eF450 | eBioscience 48-0037-42 |
|  | CD4 | OKT4 | BV711 | Sony Biotechnology 2187200 |
|  | CD8 | B9.11 | AF700 | Beckman Coulter B49181 |
|  | CD27 | L128 | BV510 | BD Biosciences 563092 |
|  | CD45R0 | UCHL1 | PE-Cy7 | BD Biosciences 337168 |
|  | CXCR5 | J252D4 | PErCP-Cy5.5 | BioLegend 356910 |
|  | CD56 | B159 | PE-CF594 | BD Biosciences 562289 |
|  |  |  |  |  |
| **Panel 3** | CD14 | RMO52 | ECD | Beckman Coulter IM2707U |
|  | CD16 | 3G8 | BV785 | BioLegend 302046 |
|  | CD11b | ICRF44 | AF700 | BD Biosciences 557918 |
|  | CD62L | DREG-56 | BV650 | BioLegend 304832 |
|  | CD203c | NP4D6 | PE-Cy7 | eBioscience 25-2039-41 |
|  | CD117 | 104D2 | PerCP-Cy5.5 | BioLegend 313214 |
|  | FCεRI | AER-37 | PB | BioLegend 334618 |
|  |  |  |  |  |
| **Panel 4** | -CD3 (Lineage) | SP34-2 | V500 | BD Biosciences 560770 |
|  | -CD19 (Lineage) | HIB19 | BV510 | BioLegend 302242 |
|  | -CD56 (Lineage) | HCD56 | BV510 | Sony Biotechnology 2191700 |
|  | HLA-DR | G46-6 | BV605 | BD Biosciences 562845 |
|  | BDCA1 (CD1c) | L161 | BV421 | BioLegend 331526 |
|  | BDCA2 (CD303) | 201A | PerCP-Cy5.5 | Sony Biotechnology 2371050 |
|  | BDCA3 (CD141) | 1A4 | BV711 | BD Biosciences 563155 |
|  | CD14 | RMO52 | ECD | Beckman Coulter IM2707U |
|  | CD16 | 3G8 | BV785 | BioLegend 302046 |
|  | CD11C | 3.9 | AF700 | eBioscience 56-0116-42 |
|  |  |  |  |  |
| **Additional mAbs** | LAIR-1 | DX26 | PE | BD Biosciences 550811 |
|  | SIRL-1 | 1A5 | FITC | own production |
|  |  |  |  |  |
