# Supplementary material for: Differential isoform expression of Allergin‐1 during acute and chronic inflammation
Source: Immun Inflamm Dis. 2022 Nov 25;10(12):e739. doi: 10.1002/iid3.739 (PMC9695092; doi:10.1002/iid3.739)
Supplement: Supplementary file 5 — Supporting information. [file IID3-10-e739-s005.docx]

**Supplementary Table 2. Patient characteristics and ongoing treatment.**SLEDAI: SLE disease activity index, dsDNA: double-stranded DNA, ENA: extractable nuclear antigens, Sm: Smith antigen, SS-A: Sjoegren Syndrome Antigen A, SS-B: Sjoegren Syndrome Antigen B, PM-Scl: Polymyositis-Scleroderma, RibP: Ribosomal antigen P, HCQ: Hydroxychloroquine, Aza: Azathioprine, Pred: Prednisone, MMF: Mycophenolate Mofetil, MTX: Methotrexate

| Age | Sex | SLEDAI | dsDNA (IU/mL) | ENA | Medication | History of nephritis |
| --- | --- | --- | --- | --- | --- | --- |
| 37 | F | 2 | 90 | SS-A, nRNP/Sm, histones, nucleosomes, PM-Scl | HCQ, Pred (7,5 mg), MMF | yes |
| 37 | F | 0 | 7.2 | none | HCQ, Pred (17,5 mg) | yes |
| 44 | F | 0 | <0,5 | PM-Scl, AMA-M2 | HCQ, Pred (5 mg), Aza | no |
| 49 | F | 0 | <0.5 | Sm, nRNP/Sm, nucleosomes, PM-Scl | HCQ Pred (7,5 mg), Aza | yes |
| 33 | F | 0 | 1.6 | SSA-60, nRNP/Sm | HCQ Pred (7,5 mg), Aza | yes |
| 47 | M | 0 | 1.7 | SS-A | HCQ | no |
| 52 | F | 0 | 2.9 | SS-A, SS-B | Pred (2,5 mg), MMF | yes |
| 20 | F | 8 | 121 | Sm, nRNP/Sm, nucleosomes, RibP | HCQ, Pred (10 mg), Aza | no |
| 46 | F | 0 | <0.5 | SS-A | HCQ, Pred (2,5 mg), Aza | yes |
| 54 | F | 0 | 58 | none | HCQ, Pred (5 mg), MMF | yes |
| 22 | F | 0 | 18 | SS-A, RibP | HCQ, Pred (5 mg), Aza | no |
| 55 | F | 0 | 31 | none | HCQ | no |
| 48 | F | 2 | 0.8 | SS-A | HCQ | no |
