# Supplementary material for: Differential isoform expression of Allergin‐1 during acute and chronic inflammation
Source: Immun Inflamm Dis. 2022 Nov 25;10(12):e739. doi: 10.1002/iid3.739 (PMC9695092; doi:10.1002/iid3.739)
Supplement: Supplementary file 6 — Supporting information. [file IID3-10-e739-s003.docx]

**Supplementary Figure 1. Gating strategy for immunophenotyping.** The gating strategy that was used to identify leukocyte subsets in the flow cytometry data is shown for a representative healthy donor. Leukocyte subsets were identified as follows: (A) neutrophils, CD14− CD16+, basophils CD14− CD16− CD117− FcεRI+; (B) total B cells, CD19+, naïve B cells, CD19+ CD27− IgD+, plasmablasts, CD19+ CD27+ IgD−; (C) CD4+ effector memory T cells, CD3+ CD4+ CD27− CD45R0+, CD4+ central memory T cells, CD3+ CD4+ CD27+ CD45R0+, CD4+ naïve T cells, CD3+ CD4+ CD27+ CD45R0−, CD4+ terminal effector T cells, CD3+ CD4+ CD27− CD45R0‐; (D) CD8+ effector memory T cells, CD3+ CD8+ CD27− CD45R0+, CD8+ central memory T cells, CD3+ CD8+ CD27+ CD45R0+, CD8+ naïve T cells, CD3+ CD8+ CD27+ CD45R0−, CD8+ terminal effector T cells, CD3+ CD8+ CD27− CD45R0−; (E) natural killer (NK) cells, CD3− CD56+, NKT‐like cells, CD3+ CD56+; (F) classical monocytes, CD3− CD19− CD56− HLA‐DR+ CD14++ CD16−, intermediate monocytes, CD3− CD19‐ CD56‐ HLA‐DR+ CD14+ CD16+, non‐classical monocytes, CD3− CD19− CD56‐ HLA‐DR+ CD14− CD16++; (G) myeloid DCs (mDCs), CD3− CD19− CD56− HLA‐DR+ CD11c+ BDCA1+, plasmacytoid DCs (pDCs), CD3− CD19− CD56− HLA‐DR+ CD11c− BDCA2+.

**Supplementary Figure 2. Overview of samples used in this study.** This figure illustrates the origin of the samples and the data generated with them as used in the current study. Also listed are the Figures in which the data is presented. Created with BioRender.

**Supplementary Figure 3. Allergin‐1 isotype expression on blood and airway neutrophil of two additional RSV bronchiolitis patients.** Blood and aspirated airway‐infiltrated leukocytes obtained from two intubated pediatric RSV bronchiolitis patients (in addition to the patient presented in Figure 3) were stained for cell lineage markers, LAIR‐1, SIRL‐1, and the Allergin‐1S1 and Allergin‐1S2 isoforms. Grey plots represent isotype‐matched control Ig (cIg).
